# CaMKII regulates the proteins TPM1 and MYOM2 and promotes diacetylmorphine-induced abnormal cardiac rhythms

**DOI:** 10.1038/s41598-023-32941-6

**Published:** 2023-04-10

**Authors:** Min Ji, Liping Su, Li Liu, Mengjie Zhuang, Jinling Xiao, Yaling Guan, Sensen Zhu, Lijuan Ma, Hongwei Pu

**Affiliations:** 1 School of Basic Medicine, Xinjiang Medical University, Urumqi, 830017 China; 2 Pathology, First Affiliated Hospital, Xinjiang Medical University, Urumqi, 830054 China; 3 Department of Academic Construction, First Affiliated Hospital, Xinjiang Medical University, Urumqi, 830054 China

**Keywords:** Kinases, Proteomics, Biochemistry, Cell biology, Anatomy, Cardiology, Medical research, Molecular medicine

## Abstract

Although opioids are necessary for the treatment of acute pain, cancer pain, and palliative care, opioid abuse is a serious threat to society. Heroin (Diacetylmorphine) is the most commonly abused opioid, and it can have a variety of effects on the body's tissues and organs, including the well-known gastrointestinal depression and respiratory depression; however, there is little known about the effects of diacetylmorphine on cardiac damage. Here, we demonstrate that diacetylmorphine induces abnormal electrocardiographic changes in rats and causes damage to cardiomyocytes in vitro by an underlying mechanism of increased autophosphorylation of CaMKII and concomitant regulation of myocardial contractile protein TPM1 and MYOM2 protein expression. The CaMKII inhibitor KN-93 was first tested to rescue the toxic effects of heroin on cardiomyocytes in vitro and the abnormal ECG changes caused by heroin in SD rats, followed by the TMT relative quantitative protein technique to analyze the proteome changes. Diacetylmorphine causes increased phosphorylation at the CaMKII Thr287 site in myocardium, resulting in increased autophosphorylation of CaMKII and subsequent alterations in myocardial contractile proteins, leading to myocardial rhythm abnormalities. These findings provide a theoretical basis for the treatment and prevention of patients with arrhythmias caused by diacetylmorphine inhalation and injection.

## Introduction

Poppy derivatives have been used for thousands of years, and the World Health Organization has proposed opioids as essential drugs for the treatment of acute pain, cancer pain, and palliative care^1^. Bayer resynthesized diacetylmorphine and marketed it as heroin in the nineteenth century for use in patients suffering from severe chronic pain or terminal illness^2,3^. However, due to diacetylmorphine's high biological toxicity and addictive nature, its abuse has caused significant social harm^4–7^. Diacetylmorphine has varying degrees of effect on various tissues and organs in the body, with the most serious side effects being respiratory depression and gastrointestinal depression^8–10^. It has also been demonstrated that diacetylmorphine can cause abnormal electrocardiographic parameters^11^; however, little is known about the mechanisms by which diacetylmorphine causes myocardial rhythm abnormalities.

Calcium (Ca^2+^)/calmodulin (CaM)-dependent kinase (CaMK)II is a serine/threonine (Ser/Thr)-specific phosphokinase that is predominantly the δ isoform in the heart. It can respond to changes in intracellular Ca^2+^ in cardiomyocytes^12^, and Ca^2+^ is an important second messenger in cardiomyocytes, involved in membrane excitation and myogenic fiber contraction^13^. CaMKII has a significant impact on excitation–contraction coupling in cardiac myocytes^14^, sustained activation of CaMKII plays a key role in arrhythmias, heart failure, cardiac ischemia–reperfusion and sudden cardiac death^15–17^. However, it is unknown whether CaMKII and its related downstream genes play a role in the process of diacetylmorphine-induced myocardial rhythm abnormalities. The goal of this study was to identify potential mechanisms of diacetylmorphine-induced myocardial rhythm abnormalities.

## Materials and methods

### Chemicals

Anti-CaMKIIδ (Item No. sc-100362) was purchased from Santa Cruz (USA), anti-CaMKII (phospho T287) (Item No. ab182647), anti-β Tubulin (Item No. ab6046), anti-cardiac troponin T (Item No. ab209813) were purchased from Abcam (Cambridge, UK), Tropomyosin-1/3 (Item No. D17B8) were purchased from Cell Signaling Technology (Beverly, MA, USA), Anti-MYOM2 (Item No. A20526) was purchased from ABclonal (Wuhan, China). KN-93 and KN-92 were obtained from Selleck (Houston, TX, United States).

### Ethical statement

All rat studies were conducted in accordance with approved guidelines, and all study protocols were reviewed and approved by the Medical Ethics Committee for Animal Experiments of the First Affiliated Hospital of Xinjiang Medical University (K201907-06).

### Animal studies

The animals used in this study were SD rat mammary rats as well as 40 SD rats. The SD rats were all male, 8 weeks old, with an initial weight of 210 ± 10 g. The experiment consisted of three groups of SD rats: Vehicle group, HE group, and HE + KN-93 group. The Vehicle group was injected subcutaneously with saline, the HE group was injected subcutaneously with diacetylmorphine, and the HE + KN-93 group was injected subcutaneously with diacetylmorphine and with KN-93 reagent in the tail vein, and the rats were subjected to a naloxone hydrochloride prodrome test on day 20, and each rat was observed for 30 min. Electrocardiograms were performed after administration of pentobarbital sodium anesthesia on day 50, followed by execution of the rats using the cervical dislocation method. All experimental rats were housed in individually ventilated cages with free access to food and free access to water. All animal experiments were performed in accordance with ARRIVE guidelines. All methods were performed in accordance with relevant guidelines and regulations.

### Isolation and culture of primary SD rat cardiomyocytes

Three-day-old SD rat neonatal rats were anesthetized and their skin was disinfected with 75% ethanol under aseptic conditions before their hearts were curved and clipped into D-Hanks solution. The atrial portion was cut with ophthalmic scissors, and the apical portion was divided into 7–8 flaps before being placed in trypsin and shaken overnight at 4 °C. After the digestion was completed, the supernatant was discarded, type II collagenase was added, and the mixture was shaken for 10 min at 37 °C in a water bath. The supernatant was transferred into a centrifuge tube and centrifuged at 1000 rpm for 5 min before being discarded, plus complete medium containing 5-Brdu, mixed by slow blowing, and transferred into a cell culture dish. Following differential apposition, the cells were transferred to a 37 °C cell incubator for further experiments.

### Cell processing

Diacetylmorphine was dissolved in PBS solution and used at a final concentration of 100 µmol/L. The CaMKII inhibitor KN-93 and KN-93 inactive analogue KN-92 were dissolved in dimethyl sulfoxide (DMSO) at a concentration of 1 mM/L, and both were used at a concentration of 1 μmol/L.

### Measurement of spontaneous beating frequency of cardiac myocytes

Cardiomyocytes were seeded in polylysine-treated 6-well plates at a density of 1 × 10^5^ cells/mL in a liquid volume of 2 mL per well. Following a incubation 5–7 days, four fields of view were randomly selected for each well after drug intervention, and two people were observed simultaneously under an inverted microscope, with the number of cell pulsations within 1 min being recorded and repeated eight times.

### GOT and LDH activity assay

LDH and GOT activity in the supernatant was measured using commercially available LDH assay kits (Solarbio, Beijing, China) and GOT assay kits (Solarbio, Beijing, China) according to the manufacturer's instructions to assess cell damage.

### 5,5′,6,6′-Tetrachloro-1,1′,3,3′-tetraethylbenzimidazolocarbo-cyanine iodide (JC-1) staining

Following JC-1 staining (cat. no. C2006; Beyotime Institute of Biotechnology), incubate at 37 °C for 25 min, wash twice with JC-1 staining buffer, photograph and observe under laser confocal microscope, and analyze the data using ImageJ to respond to mitochondrial membrane potential changes by the red-green fluorescence ratio.

### Western blotting

Cell precipitates and animal tissues were collected from each group. Total protein was extracted from RIPA lysates and protein concentration was measured using the BCA protein quantification method. The protein loading volume was 40 μg. Electrophoresis; transfer to PVDF membrane using the wet transfer method, close for 2 h, for a better antibody binding, western blotting images trimming of the corresponding target protein was trimmed according to the location of marker, add primary antibody, primary antibodies include CaMKIIδ (dilution ratio: 1:500), p-CaMKII (T287) (dilution ratio: 1:500), β Tubulin (dilution ratio: 1:1500), Tropomyosin-1/3 (dilution ratio: 1:1000), MYOM2 (dilution ratio: 1:1000), and shake overnight at 4 °C. Separately, goat anti-mouse IgG (1:2000) and goat anti-rabbit IgG (1:2000) were added and shaken at room temperature for 120 min. The film is washed with TBST before strip exposure. The grayscale values of each band were measured using the grayscale analysis tool included in the image Lab software, and the target protein's relative expression was compared to the grayscale value of the internal reference -tubulin to obtain the target protein's relative expression for statistical analysis.

### Electrocardiogram testing

Rats were anesthetized with 2% pentobarbital sodium in the supine position. Using the BL-420 Biofunctional Experiment System, a silver needle was inserted into the rats' right forelimb, left hindlimb, and right hindlimb, and then the white alligator clip was connected to the silver needle of the right forelimb, the red alligator clip to the silver needle of the left hindlimb, and the black alligator clip to the silver needle of the right hindlimb. Recording of standard II-lead ECG.

### Tandem mass spectrometry labeling for relative quantitative proteomics screening of differentially expressed proteins

Following cell extraction, quantification, TMT labeling, peptide grading, data acquisition using liquid chromatography-tandem mass spectrometry (LC–MS/MS), and finally screening for differentially expressed proteins, each group of cells was processed (this part of the experiment was done by Shanghai Zhongke New Life Biotechnology Co.).

### Volcano map

To screen differentially expressed proteins, we calculated Fold change and P-value from t-test using the criteria of Fold change greater than 1.2-fold (up-regulation greater than 1.2-fold or down-regulation less than 0.83) and P-value less than 0.05. The horizontal coordinates represent the Fold change (log-transformed by 2), and the vertical coordinates represent the difference's P-value. The horizontal coordinate is the number of fold differences (log-transformed by 2), the vertical coordinate is the difference's P-value (log-transformed by 10), and the volcano plot is created in R with "ggplot."

### Venn diagram

After excluding undefined proteins, common factors were screened between drug vs con up-regulation factors and KN-93 vs drug down-regulation factors, and between drug vs con down-regulation factors and KN-93 vs drug up-regulation factors, using the "VennDiagram" in R to generate Venn diagrams.

### GO functional enrichment analysis

Gene Ontology (http://www.geneontology.org/) is a standardized functional classification system that provides a dynamically updated standardized vocabulary and describes the properties of genes and gene products in an organism in three aspects: Biological Process (BP), Molecular Function (MF), and Cellular Component (CC). We used Blast2Go (https://www.blast2go.com/) software for GO functional annotation of all identified proteins, followed by Fisher's exact test GO functional enrichment analysis of differentially expressed proteins.

### KEGG pathway enrichment analysis

One of the most commonly used databases for pathway research is KEGG (Kyoto Encyclopedia of Genes and Genomes, http://www.kegg.jp/). The KEGG database contains information on metabolic pathways, genetic data processing, environmental data processing, cellular processes, organismal systems, human disease development, and other topics. Information. The KEGG pathway enrichment analysis analyzes and calculates the significance level of protein enrichment for each pathway using the KEGG pathway as the unit and the total proteins identified as the background, and the Fisher's Exact Test to identify the significantly affected metabolic and signal transduction pathways.

### Hierarchical clustering, heatmap visualization

A hierarchical clustering heatmap is generated in R using the "ComplexHeatmap" package based on the factors sieved from the Wayne diagram.

### Protein interaction network diagram

The STRING (http://string-db.org/) database was used to discover the interactions between the target proteins, and the CytoScape software was used to generate the interaction networks.

### Statistical analysis

The results are presented as mean SD, and the data was evaluated using a t-test on two independent samples. ANOVA with LSD post hoc test for multiple comparisons, chi-square test for rate comparisons, SPSS 26 software for data analysis, and GraphPad 8.0.2 software for graphing were used to analyze multiple data sets. A statistically significant p-value of 0.05 was used.

## Results

### CaMKII inhibitors attenuate the toxic effects of diacetylmorphine on cardiomyocytes in vitro

**Figure 1:**
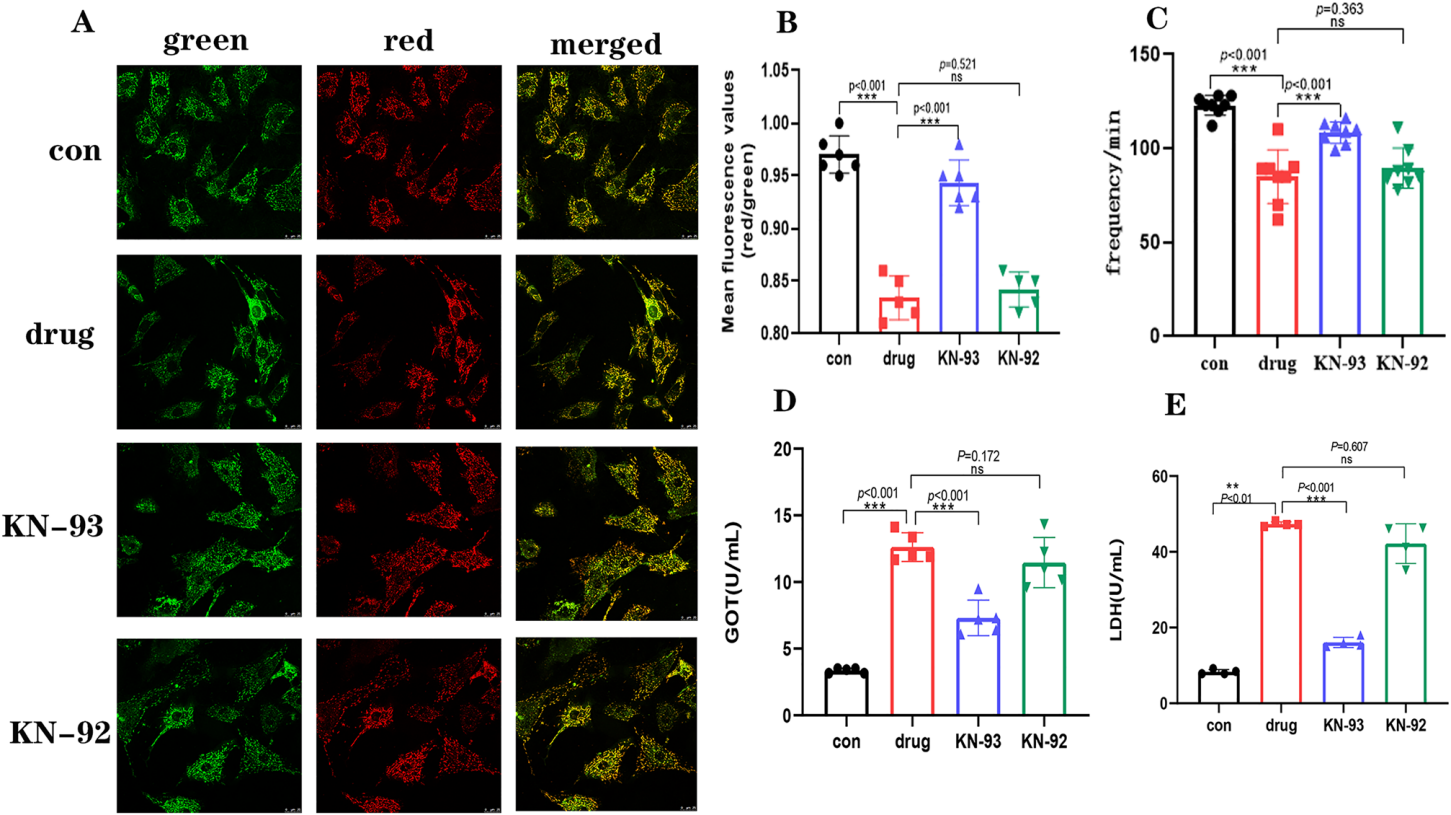
Effect of KN-93 on diacetylmorphine-induced cardiomyocyte injury in vitro. (**A**) Representative immunofluorescence images of JC-1 fluorescent probe showing the effects of diacetylmorphine and KN-93 and KN-92 on the mitochondrial membrane potential of cardiomyocytes. (**B**) Quantitative plot of A, mean ± SD. (**C**) Histogram of cardiomyocyte beat frequency for each group, mean ± SD. (**D**–**E**) LDH and GOT activity detection. All data are expressed as mean ± SD, *P < 0.05, **P < 0.01, ***P < 0.001, ****P < 0.0001, ns: *P* > 0.05.

We cultured primary SD rat neonatal rat cardiomyocytes in vitro and identified cardiomyocytes using Anti-cardiac troponin T (Supplemental Instrument Supplementary Fig. 1), which showed a purity of > 95% and could be used for subsequent experiments. Except for the cell control group(con), they were divided into the drug group (treated with 100 µmol/L heroin for 24 h), KN-93 group (treated with 100 µmol/L heroin + 1 μmol/L KN-93 for 24 h) and KN-92 group (100 µmol/L heroin + 1 μmol/L KN-92 for 24 h). Cardiomyocytes cultured for about 5–7 days resumed beating with a beating frequency averaging 120 beats/min. The beating frequency decreased significantly after diacetylmorphine treatment, averaging at 81 beats/min, whereas with KN-93, the beating frequency increased compared to the drug group, and there was no statistical difference between the KN-92 and drug groups (Fig. 1C). To observe the toxic effects of CaMKII inhibitor to attenuate KN-93 diacetylmorphine on cardiomyocytes in vitro in a multifaceted manner, we used JC-1 fluorescent probe and observed the mitochondrial membrane potential changes in each group using laser confocal microscope, and the results showed that the mitochondrial membrane potential was decreased in the drug group compared with the con group, while it was increased in the KN-93 group compared with the drug group, and there was no significant change in the KN-92 group (Fig. 1A,B). Subsequently, we measured the LDH and GOT levels in the cell culture supernatant and the results showed that the LDH and GOT levels in the drug group were higher than those in the con group, while the LDH and GOT levels in the KN-93 group were lower than those in the drug group, and there was no significant change in the LDH and GOT levels in the KN-92 and drug groups (Fig. 1DE). These findings suggest that KN-93 reduces the toxic effects of diacetylmorphine on cardiomyocytes in vitro. It also suggests that CaMKII is involved in the toxic effects of diacetylmorphine on cardiomyocytes in vitro.

### Diacetylmorphine regulates cardiomyocyte protein expression patterns

**Figure 2:**
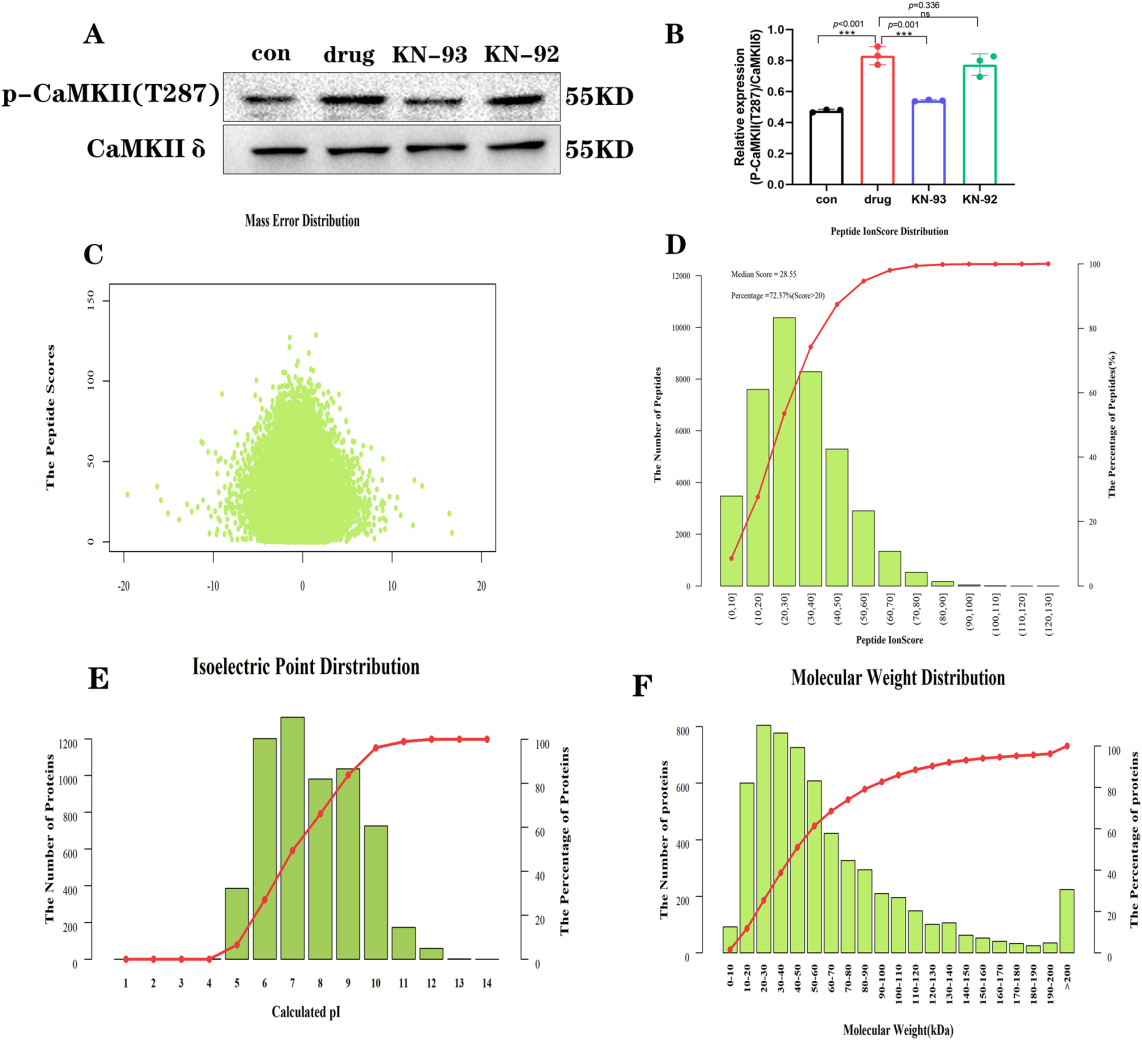
Effect of diacetylmorphine and KN-93 and KN-92 on CaMKII phosphorylation in cardiomyocytes and a quality control note on proteomics. (**A**) Protein blot analysis of P-CaMKII (T287) in each group, original blots are presented in Supplementary Information Fig. 1, samples derive from the same experiment and that blots were processed in parallel. Quantification is shown in (**B**). (**C**) Peptide ion mass deviation distribution. (**D**) Peptide ion score distribution. (**E**) The horizontal coordinate is the isoelectric point of the identified proteins; the main vertical coordinate is the distribution of isoelectric points of proteins. The histogram in the graph corresponds to the number of proteins identified with the corresponding isoelectric points; the secondary vertical coordinate corresponds to the cumulative curve in the graph, indicating the cumulative percentage of proteins with isoelectric points not higher than the corresponding value. (**F**) The distribution of relative molecular masses of proteins, the horizontal coordinate is the relative molecular weight of the identified proteins; the main vertical coordinate Number of proteins corresponds to the histogram in the figure, indicating the number of proteins with the corresponding relative molecular masses identified; the secondary vertical coordinate corresponds to the cumulative curve in the figure, indicating the cumulative percentage of proteins with no higher than the corresponding relative molecular masses.

To ascertain the effect of diacetylmorphine on cardiomyocytes as well as the mechanism of action of the CaMKII protein in it. We exposed cardiomyocytes to diacetylmorphine at a concentration of 100 μmol/L and either KN-93 at a concentration of 1 μmol/L or KN-92 at a concentration of 1 μmol/L. We first used protein blotting to detect the phosphorylation level of CaMKIIδ protein and its T287 site, and the results showed that the phosphorylation level was significantly higher in the drug group compared to the control group, while it was lower in the KN-93 group (Fig. 2A,B). We used an unbiased screening method and performed proteomic analysis with samples from the con group, drug group, and KN-93 group, with three biological replicates in each group, to further determine the mechanism of action of heroin on cardiomyocytes and the molecular mechanism of CaMKII protein in it. The mass deviation of all identified peptides was mostly within 10 ppm (Fig. 2C), and the distribution of peptide ion scores: approximately 72.37% of the peptides scored above 20, and the median score of peptides was 28.55 (Fig. 2D), indicating that the identification results were accurate and reliable, and the distribution of the identified protein characteristics was shown in Fig. 2E,F.

**Figure 3:**
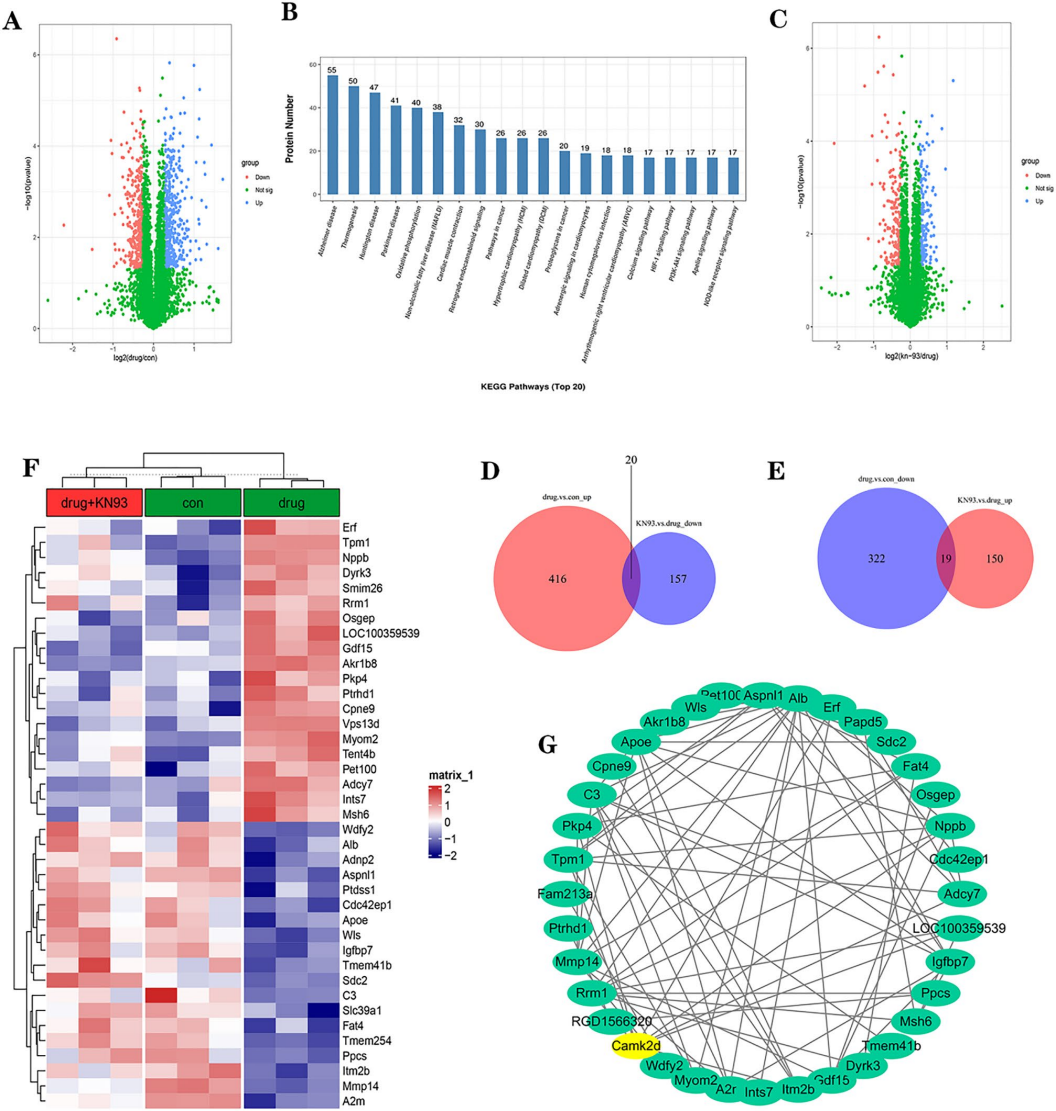
Bioinformatics analysis identified differentially expressed proteins and enriched pathways. (**A**) Drug vs con differentially expressed proteins (DEGs-1), red dots are significantly down-regulated and blue dots are significantly up-regulated. (**B**) KEGG pathways Top20 (DEGs-1), (**C**) KN-93 vs drug differentially expressed proteins (DEGs-2), red dots are significantly down-regulated and blue dots are significantly up-regulated. (**D**) Drug vs con upregulation of differential proteins in common with KN-93 group vs drug downregulation of differential proteins, presented in a Venn diagram. DEGs-1 upregulation in common with DEGs-2 downregulation, presented in a Venn diagram. (**E**) Common proteins of Drug vs con down-regulated differential proteins with KN-93 group vs drug up-regulated differential proteins, presented in a Wayne diagram. (**F**) Includes 9 samples with 3 replicates per group, grouped into con, drug, and KN-93 groups. Each row in the heat map represents a protein whose expression is normalized across columns, with high expression shown in red and low expression shown in blue. (**G**) Protein interaction map of 39 proteins with CaMKIIδ.

A total of 784 differentially expressed proteins were screened in the drug vs con group using the criteria of ploidy change greater than 1.2-fold (up-regulation greater than 1.2-fold or down-regulation less than 0.83) and P value less than 0.05. These differentially expressed proteins were defined as DEGs-1, of which 442 were up-regulated differentially expressed proteins and 342 were down-regulated proteins (Fig. 3A). As shown in Table 1: DEGs-1 was mainly involved in cardiac muscle tissue development, actin binding and other processes, while sarcomere, I band and other sites of protein were significantly altered. As shown in Fig. 3B, KEGG pathways such as Cardiac muscle contraction and Retrograde endocannabinoid signaling were changed more significantly (Fig. 3B).

**Table 1:** Enriched GO terms (top 20).

GO_ID	Term	Rich factor	P value	Classify
GO:0055001	Muscle cell development	0.465	0.000	BP
GO:0055002	Striated muscle cell development	0.467	0.000	
GO:0061061	Muscle structure development	0.300	0.000	
GO:0048738	Cardiac muscle tissue development	0.402	0.000	
GO:0014706	Striated muscle tissue development	0.341	0.000	
GO:0060537	Muscle tissue development	0.333	0.000	
GO:0051146	Striated muscle cell differentiation	0.360	0.000	
GO:0003015	Heart process	0.409	0.000	
GO:0050136	NADH dehydrogenase (quinone) activity	0.739	0.000	MF
GO:0008137	NADH dehydrogenase (ubiquinone) activity	0.739	0.000	
GO:0016655	Oxidoreductase activity, acting on NAD(P)H, quinone or similar compound as acceptor	0.625	0.000	
GO:0003954	NADH dehydrogenase activity	0.708	0.000	
GO:0003779	Actin binding	0.270	0.000	
GO:0016651	Oxidoreductase activity, acting on NAD(P)H	0.418	0.000	
GO:0030017	Sarcomere	0.441	0.000	CC
GO:0030016	Myofibril	0.420	0.000	
GO:0044449	Contractile fiber part	0.423	0.000	
GO:0043292	Contractile fiber	0.403	0.000	
GO:0031674	I band	0.466	0.000	
GO:0098803	Respiratory chain complex	0.507	0.000	

To further determine the specific molecular mechanism of CaMKII protein in the action of diacetylmorphine on cardiomyocytes. A total of 346 differentially expressed proteins were screened in the KN-93 vs drug group using the criteria of ploidy change greater than 1.2-fold (up-regulation greater than 1.2-fold or down-regulation less than 0.83) and P value less than 0.05, and these 346 differentially expressed proteins were defined as DEGs-2, of which 169 were up-regulated and 177 were down-regulated (Fig. 3C). The common proteins of up-regulated proteins in DEGs-1 and down-regulated proteins in DEGs-2 were searched for a total of 20 proteins (Fig. 3D), then look for proteins that are down-regulated in DEGs-1 in common with the up-regulated proteins in DEGs-2, a total of 19 proteins (Fig. 3E), and these 39 proteins were defined as DE-CaMKIIs. Figure 3F depicts the expression of DE-CaMKIIs in each group (Fig. 3F). The DE-CaMKIIs protein interaction network prediction with CaMKII revealed that DE-CaMKIIs interacted with CaMKIID (Fig. 3G).

### Abnormal cardiomyocyte rhythm caused by diacetylmorphine is associated with CaMKII regulation of TPM1 and MYOM2 proteins

**Figure 4:**
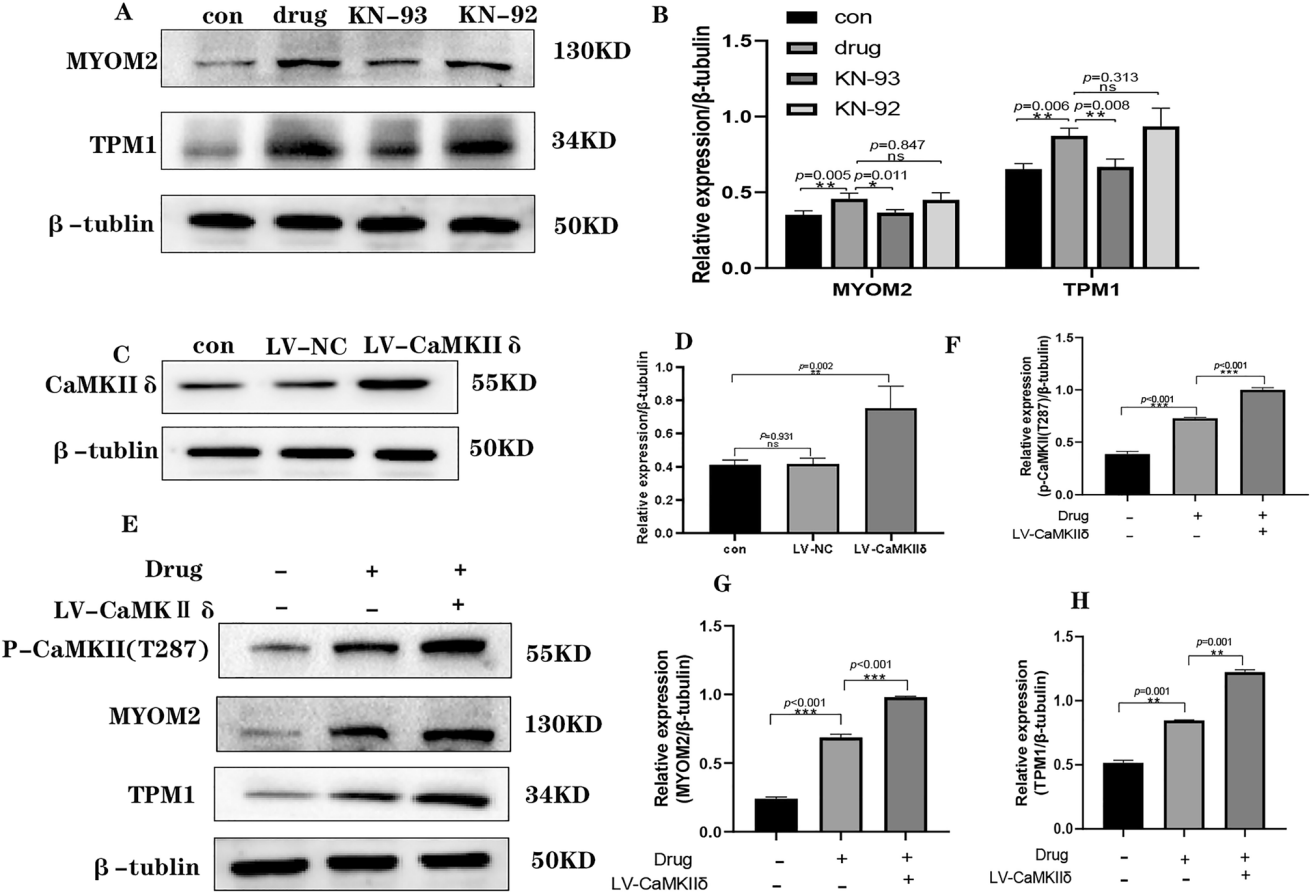
Effects of diacetylmorphine on TPM 1, MYOM 2 in cardiomyocytes. (**A**) Western blotting of TPM1, MYOM2 in cardiomyocytes of cell, original blots are presented in Supplementary Information Fig. 2, samples derive from the same experiment and that blots were processed in parallel. Quantification is in (**B**). (**C**) Western blotting of CaMKIIδ overexpression lentivirus transfection efficiency, original blots are presented in Supplementary Information Fig. 3, samples derive from the same experiment and that blots were processed in parallel. Quantification is in (**D**). (**E**) After transfection with CaMKIIδ overexpressing lentivirus, p-CaMKII, TPM1 and MYOM2 expression in cardiomyocytes was detected by western blotting using 100 µmol/L diacetylmorphine intervention, original blots are presented in Supplementary Information Fig. 4, samples derive from the same experiment and that blots were processed in parallel. Quantification in (**F**–**H**). All data are expressed as mean ± SD, *P < 0.05, **P < 0.01, ***P < 0.001, ****P < 0.0001, ns: *P* > 0.05.

To further confirm the mechanism of CaMKII in myocardial rhythm abnormalities caused by diacetylmorphine, we selected the myocardial contraction-related proteins MYOM2 and TPM1 after combining the results of previous proteomic analyses, and examined the changes in their expression levels in each group using protein blotting. We cultured primary SD rat suckling rats cardiomyocytes in vitro and exposed the cultured cells to diacetylmorphine at a concentration of 100 μmol/L as well as KN-93 at a concentration of 1 μmol/L or KN-92 at a concentration of 1 μmol/L for 24 h. Western Blot results showed that the relative expression levels of TPM1 and MYOM2 protein were significantly higher in the drug group compared with the con group, and the difference was statistically significant, while the relative expression levels of TPM1 and MYOM2 protein were lower in the KN-93 group compared with the drug group, and the difference was statistically significant, while there was no significant difference in the relative expression levels of TPM1 and MYOM2 protein in the KN-92 group compared with the drug group (Fig. 4A,B). This result is consistent with the proteomic results. To further determine the relationship between CaMKII and cardiac contractile proteins TPM1 and MYOM2, we used CaMKIIδ overexpression lentivirus to infect primary SD rat neonatal rat cardiomyocytes and verified the transfection efficiency using Western Blot, and the results showed that the relative expression level of CaMKIIδ protein in the CaMKIIδ overexpression group (LV-CaMKIIδ group) was significantly higher than that in the cell control group (con group) and the null-stained group (LV-NC group) (Fig. 4C,D). Subsequently, We treated LV-CaMKIIδ group cardiomyocytes with diacetylmorphine and detected the changes of p-CaMKII(T287), TPM1, MYOM2 protein relative expression levels using Western Blot, and the results showed that compared with the con group, the p-CaMKII(T287), TPM1, and MYOM2 protein relative expression levels were increased in the drug + LV-CaMKIIδ group, and p-CaMKII(T287), TPM1, MYOM2 protein relative expression levels were significantly increased in the drug + LV-CaMKIIδ group compared with the drug group, and the differences were statistically significant (Fig. 4E–H). The above Western Blot results all suggest that diacetylmorphine-induced rhythm abnormalities in cardiomyocytes are associated with CaMKII regulation of TPM1 and MYOM2 proteins.

### KN-93 reduces the frequency of electrocardiographic abnormalities in diacetylmorphine-addicted rats

**Figure 5:**
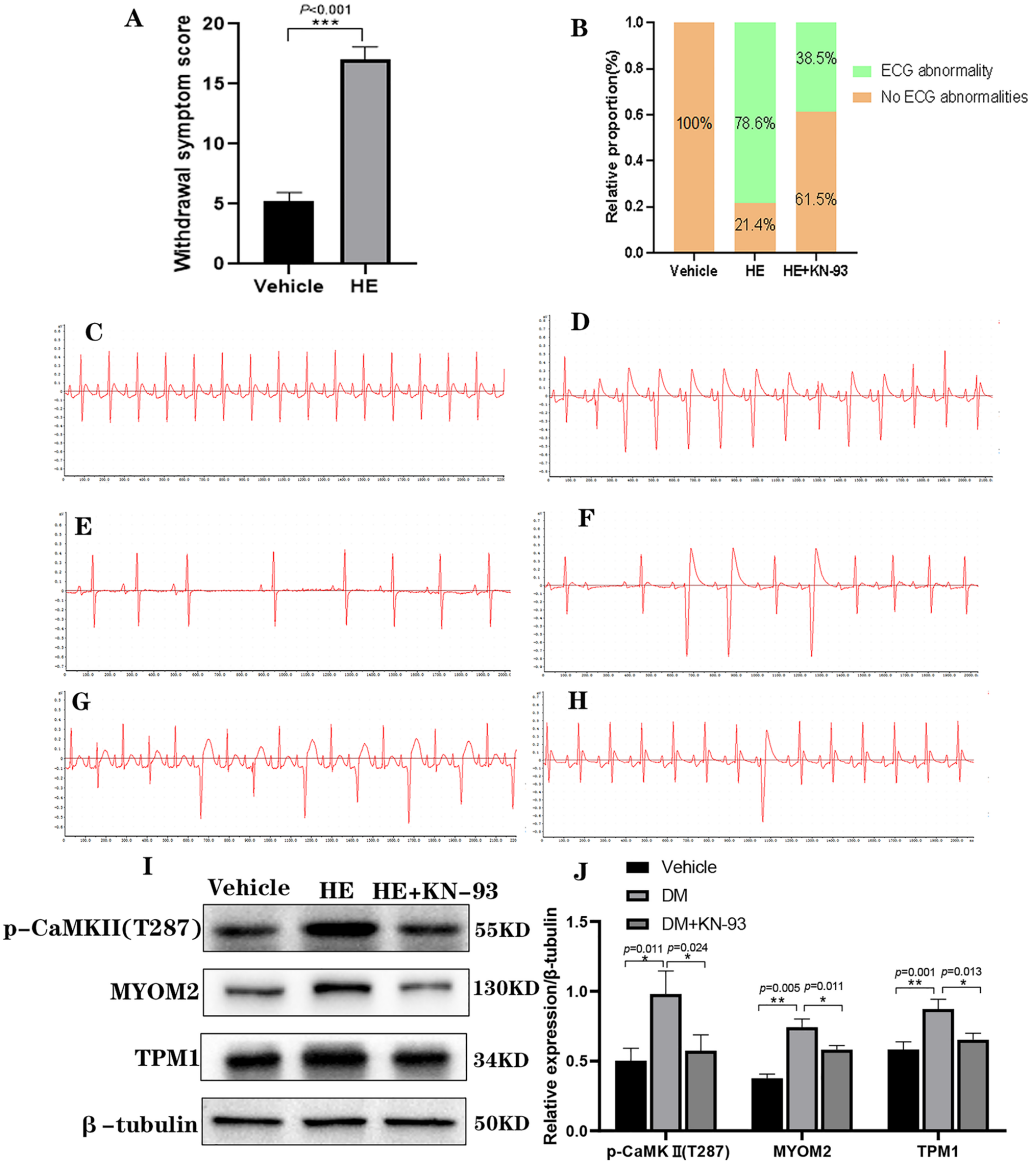
Effect of KN-93 on electrocardiogram and myocardial tissue p-CaMKII (T287), TPM1 and MYOM2 proteins in diacetylmorphine addicted rats. (**A**) Withdrawal symptom score. (**B**) Incidence of ECG abnormalities in each group of rats. (**C**) ECG of Vehicle group rats (50 ms/div). (**D**–**G**) Representative electrocardiogram of HE group rats (50 ms/div). (**H**) Representative ECG of rats in HE + KN-93 group (50 ms/div). (**I**) Western blotting of p-CaMKII(T287), TPM1, and MYOM2 in myocardial tissue of Vehicle group, HE group, and HE + KN-93 group, original blots are presented in Supplementary Information Fig. 5, samples derive from the same experiment and that blots were processed in parallel. Quantification is in (**J**).

In order to explore the effect of heroin on ECG, we established a heroin addiction model in SD rats by dose-escalation method, and scored according to withdrawal symptoms (Supplemental Instrument Supplementary Table 1) to determine whether addiction was present, and the results showed that the HE group scored significantly higher than the Vehicle group (Fig. 5A), indicating that the model was established successfully. Subsequent injection of KN-93 and observation of II-lead ECG using BL-420 showed no abnormal ECG in the Vehicle group (Fig. 5C), in contrast, various types of ECG abnormalities, such as sinus arrest, complete right bundle branch block, premature ventricular beats, and ventricular tachycardia, were observed in the SD rats of the HE group (Fig. 5D–G), and ECG abnormalities were also observed in rats in the HE + KN-93 group (Fig. 5H), but were rare compared with the HE group (Fig. 5B and Supplemental Instrument Supplementary Table 2). The changes in the relative expression levels of p-CaMKII (T287), TPM1, and MYOM2 proteins were detected using Western Blot, and the results showed that the relative expression levels of p-CaMKII (T287), TPM1, and MYOM2 proteins were increased in the HE group compared with the Vehicle group, and the relative expression levels of p-CaMKII (T287), TPM1, and MYOM2 proteins were decreased in the HE + KN-93 group compared with the HE group, and the differences were statistically significant (Fig. 5I,J). These results suggest that KN-93 reduces the frequency of ECG abnormalities in diacetylmorphine-addicted rats and also suggest that CaMKII plays an important role in diacetylmorphine-induced ECG abnormalities in rats.

## Discussion

In recent years, the mechanism of diacetylmorphine abuse on cardiovascular damage has become a hot research topic. Some studies have shown that diacetylmorphine can cause abnormal ECG parameters, and some scholars have found that diacetylmorphine addiction and abuse can cause different degrees of changes in the ECG of rats, showing various types of arrhythmias, and also diacetylmorphine can cause changes in the frequency of spontaneous beats of cardiac myocytes, but the specific mechanism is still unclear and The exact mechanism is unknown and needs to be further investigated.

Diacetylmorphine can cause different degrees of damage to various systems and organs, such as adrenal insufficiency, multi-organ function syndrome, cardiac arrest, etc.^18–20^. Here, we found that diacetylmorphine had an injurious effect on cardiomyocytes cultured in vitro, causing a decrease in spontaneous beat frequency, a decrease in mitochondrial membrane potential, and LDH and GOT release increase whereas KN-93, an inhibitor of CaMKII, had a protective effect on cardiomyocytes after diacetylmorphine intervention. KN-93 is a CaMKII-specific inhibitor that inhibits CaMKII activity^21^. In contrast, CaMKII belongs to the serine/threonine protein kinase family and is regulated by the Ca^2+^/CaM complex, with four isoforms, α, β, γ and δ. The δ isoform is mainly expressed in the myocardium. Meanwhile, CaMKII is considered to be an important factor in the excitation–contraction coupling process, which can affect the change of Ca^2+^ content in cardiac myocytes. Moderate activation is beneficial for the normal functioning of the heart, while continuous activation may lead to cardiac dysfunction. Many studies have now shown that CaMKII is closely associated with the development of cardiovascular diseases such as arrhythmia, cardiac hypertrophy and heart failure^17,22–24^.

In this study, the differentially expressed proteins and enriched pathways of diacetylmorphine intervention in cardiac myocytes were analyzed using proteomics, and it was found that differentially expressed proteins are mainly involved in muscle cell development, striated muscle cell development, muscle structure development, cardiac muscle tissue development, and other important biological processes, while cardiac muscle contraction is in the first 20 of the KEGG-enriched pathway. Marcin Kunecki et al. also demonstrated experimentally that morphine affects the systolic and diastolic functions of human heart muscle^25^. Further analysis by proteomics revealed that two contractile proteins, TPM1 and MYOM2, are likely to be involved in diacetylmorphine-induced myocardial rhythm abnormalities as CaMKII downstream proteins.

Proto-myosin (Tm) is a large family of highly conserved alpha-helix coiled-coil proteins, and TPM1 is a member of this large family^26^. Prothymosin, troponin, and myosin work together to move between blocked, closed, and open sites on thin filaments, thereby masking and exposing the actin binding sites necessary for myosin to interact across bridgeheads^27^. MYOM2, a member of the myomesin protein family, is a major component of the myofibrillar myogenic fiber M-band and a central gene in myofibrillar gene interactions^28^. In contrast, CaMKII is a signaling molecule whose activity is triggered by an increase in intracellular Ca^2+^ levels, an activity that can be sustained by enzymatic autophosphorylation, generating molecular memory concentrations after a decrease in Ca^2+^; it regulates a variety of proteins that are involved not only in ECC and relaxation but also in cell death, transcriptional activation of hypertrophy, inflammation, and arrhythmias^29,30^. We verified by Western Bloting assay and found that diacetylmorphine increased the phosphorylation level of CaMKII T287 site in cardiomyocytes and increased the relative expression of TPM1 and MYOM2 proteins compared with the con group, while after using CaMKII inhibitor KN-93, compared with the drug group, the phosphorylation level of CaMKII T287 site The relative expression of TPM1 and MYOM2 proteins was decreased. It is further suggested that myocardial rhythm abnormalities caused by diacetylmorphine are associated with CaMKII regulation of TPM1 and MYOM2 proteins.

Subsequent pharmacological interventions at the animal level revealed that heroin caused abnormal electrocardiographic changes in SD rats, whereas KN-93 reduced the occurrence of electrocardiographic abnormalities, and heroin increased the phosphorylation level of CaMKII T287 site and the relative expression of TPM1 and MYOM2 proteins in SD rat myocardial tissue, whereas KN-93 decreased the phosphorylation level of CaMKII T287 site and the relative expression of TPM1 and MYOM2 proteins in SD rat myocardial tissue.

The contraction and diastole of the myocardium is a complex process. Cardiac muscle is a transverse muscle that coordinates contraction in response to neuronal stimulation through a voltage- and calcium-dependent process of excitation–contraction coupling^31^. Myocardial contraction is mainly determined by contractile proteins (actin and troponin), regulatory proteins (troponin and prothymosin, etc.), and Ca^2+^^32^. Thus, it is not only the dysregulation of intracellular Ca^2+^ homeostasis in cardiac myocytes that affects myocardial rhythm, but also myocardial contraction-related proteins.

In conclusion, diacetylmorphine induces increased levels of phosphorylation at the myocardial CaMKII Thr287 site, which increases CaMKII autophosphorylation. which in turn causes alterations in the myocardial contractile proteins TPM1 and MYOM2, leading to abnormal myocardial rhythms. These findings provide a theoretical basis for the treatment and prevention of patients with arrhythmias caused by diacetylmorphine inhalation and injection.

## Supplementary Information

Supplementary Tables.

Supplementary Figures.

## Data Availability

The datasets generated during and/or analysed during the current study are available from the corresponding author on reasonable request.

## Abbreviations

CaMKII: Ca^2+^/calmodulin-dependent protein kinaseII
TPM1: Tropomyosin1
MYOM2: Myomesin2
Ca^2+^: Calcium
ECG: Electrocardiograph
