# Supplementary material for: CaMKII regulates the proteins TPM1 and MYOM2 and promotes diacetylmorphine-induced abnormal cardiac rhythms
Source: Sci Rep. 2023 Apr 10;13:5827. doi: 10.1038/s41598-023-32941-6 (PMC10085977; doi:10.1038/s41598-023-32941-6)
Supplement: Supplementary file 1 — Supplementary Tables. [file 41598_2023_32941_MOESM1_ESM.docx]

Supplementary table 1-Withdrawal symptom score

| Withdrawal symptom | evaluation standards | | |
| --- | --- | --- | --- |
|  | 1 | 2 | 3 |
| twister | 1~3 | 4~6 | ≧7 |
| Wet dog shake | 1~3 | 4~6 | ≧7 |
| jump | 1~3 | 4~6 | ≧7 |
| Tooth fibrillation | 1~3 | 4~6 | ≧7 |
| stand | 1~3 | 4~6 | ≧7 |
| Clean up the fur | 1~3 | 4~6 | ≧7 |
| ptosis | 1~4 | 5~8 | ≧9 |

Supplementary table 2

| grouping | Example number | An ECG abnormality occurred | No ECG abnormalities occurred | The incidence of ECG abnormalities |
| --- | --- | --- | --- | --- |
| Vehicle | 6 | 0 | 6 | 0 |
| HE | 14 | 11 | 3 | 0.785714286 |
| HE+KN-93 | 13 | 5 | 8 | 0.384615385 |
| total | 33 | 15 | 16 |  |


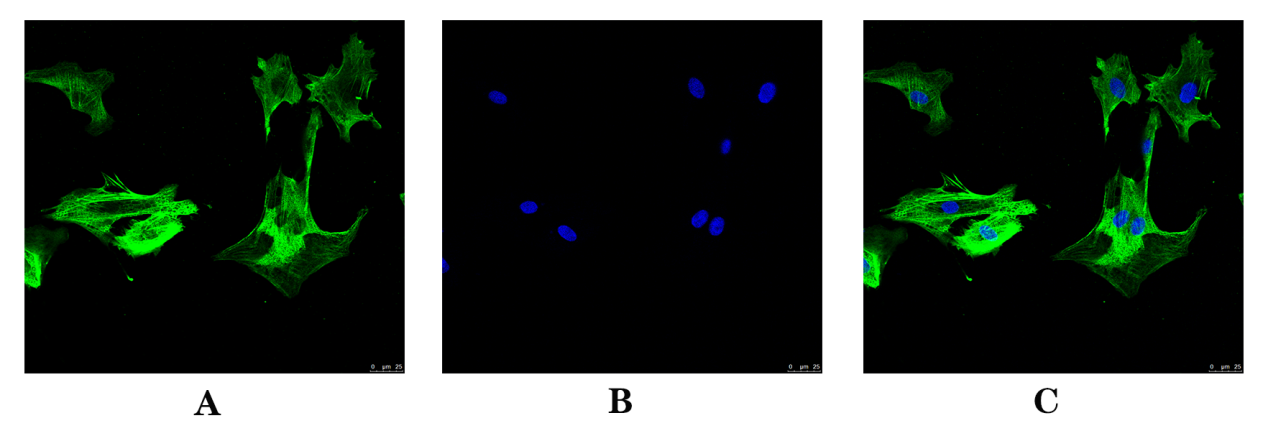


Supplementary figure 1-Immunofluorescence identification of cardiomyocytes

(A)Anti-cardiac troponin T was specifically stained in green；(B)Nuclei were stained in blue with DAPI；(C)A+B compose C
