# Supplementary material for: CaMKII regulates the proteins TPM1 and MYOM2 and promotes diacetylmorphine-induced abnormal cardiac rhythms
Source: Sci Rep. 2023 Apr 10;13:5827. doi: 10.1038/s41598-023-32941-6 (PMC10085977; doi:10.1038/s41598-023-32941-6)

Western blotting images trimming of the corresponding target protein was trimmed according to the location of marker. The original blot images used in the article are specific as follows:

Figure 1:**Fig.2-A**


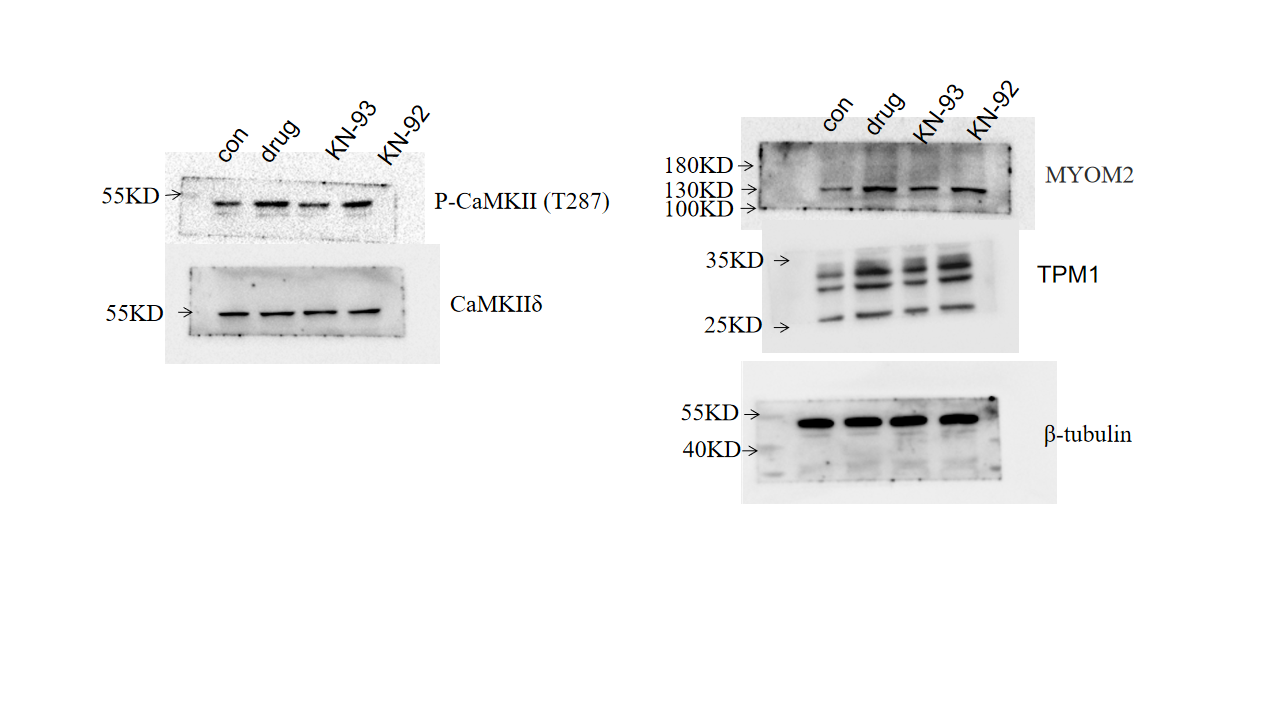


Figure 2:**Fig.4-A**

**
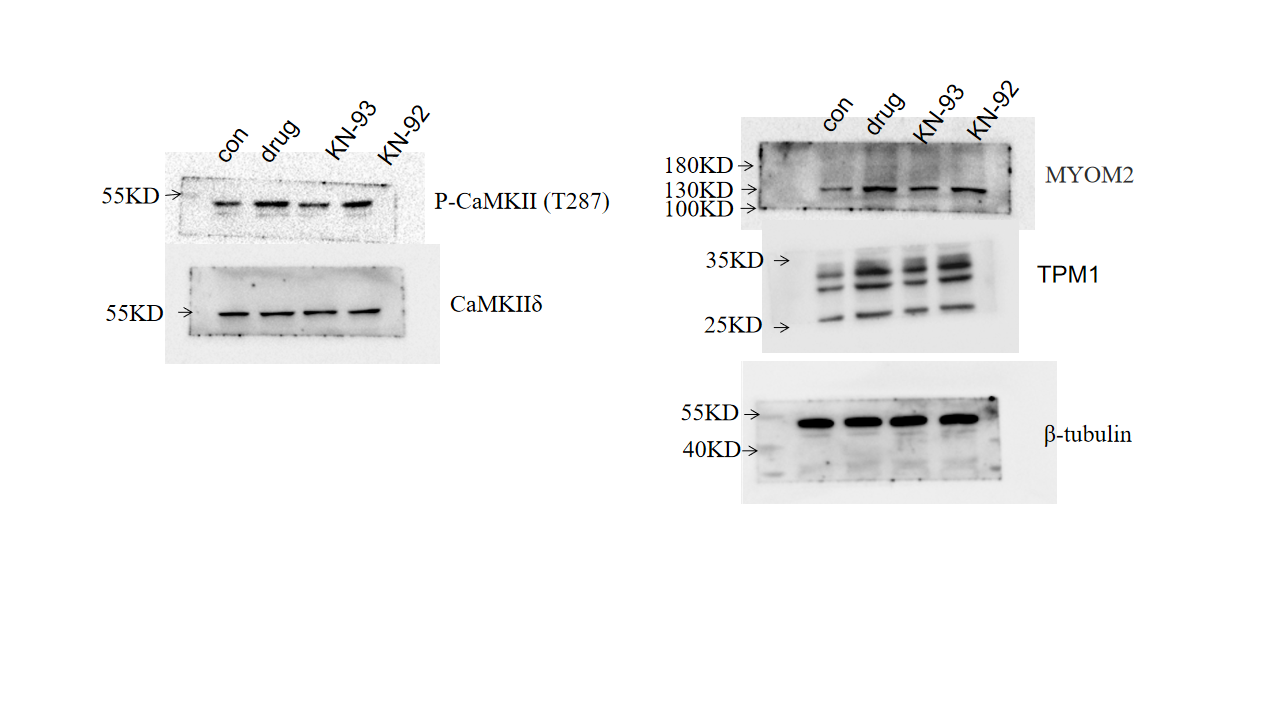
**

Figure 3:**Fig.4-C**


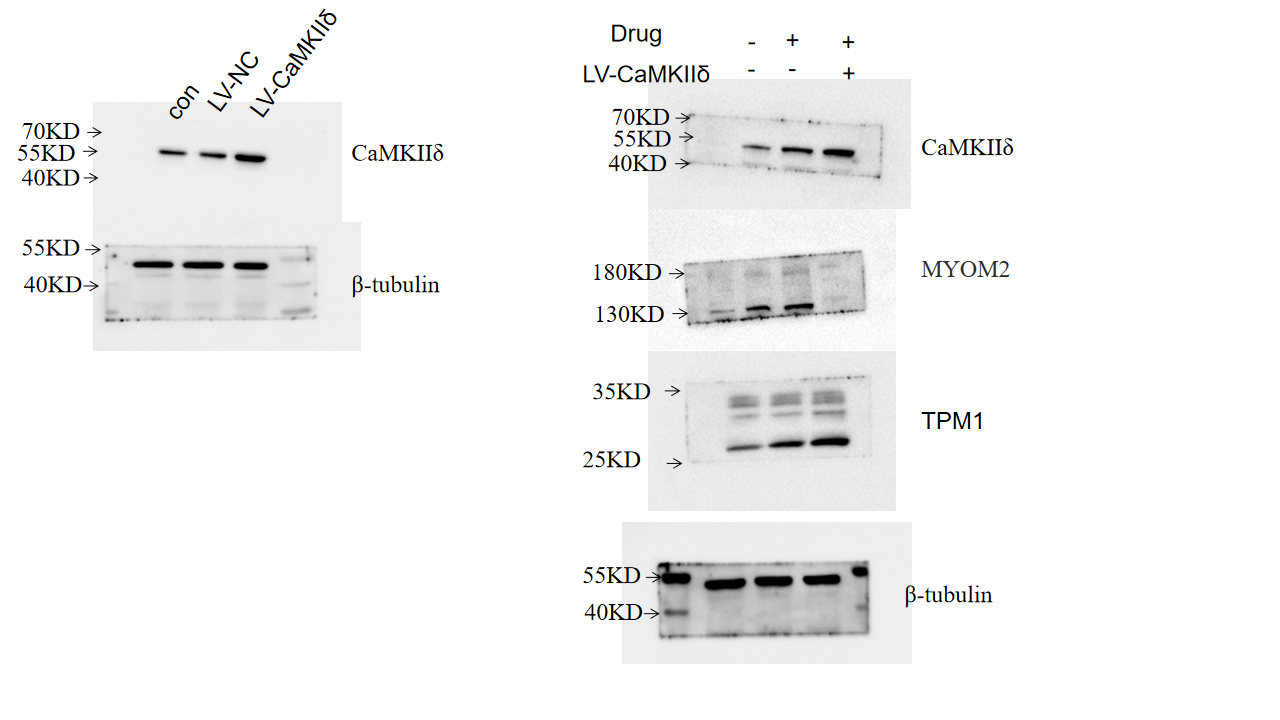


Figure 4:**Fig.4-E**

**
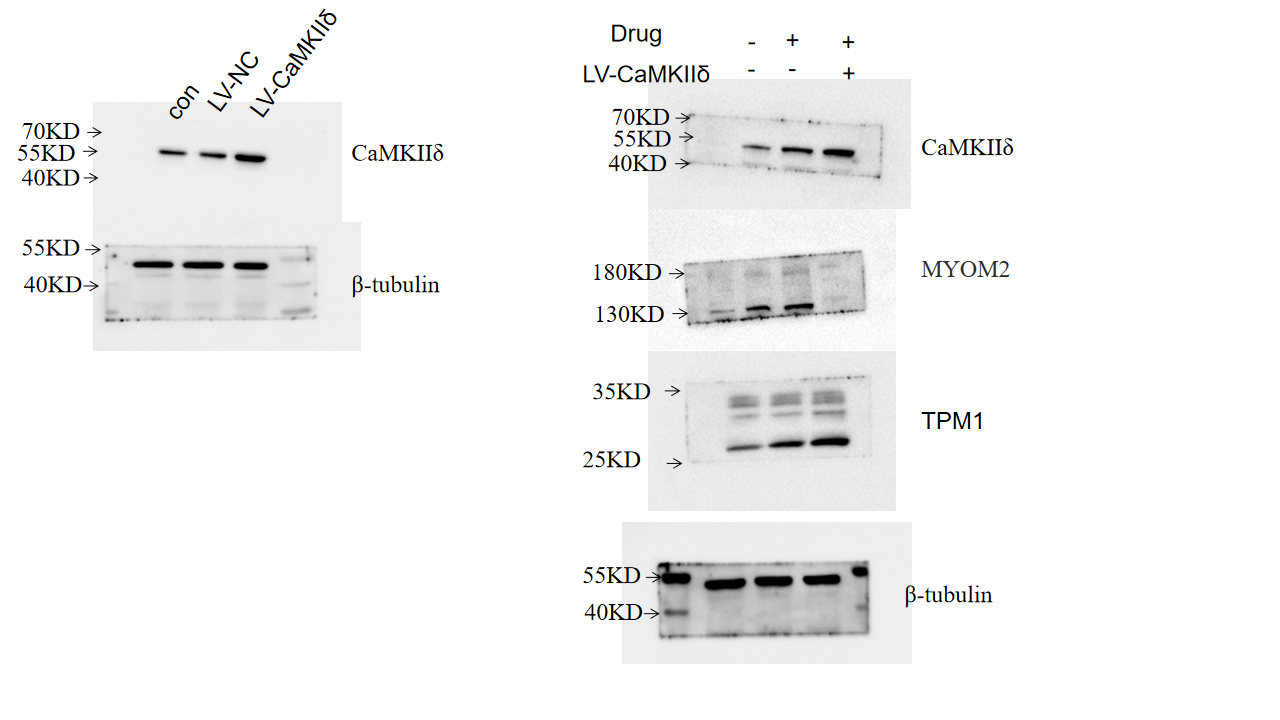
**

Figure 5:**Fig.5-I**


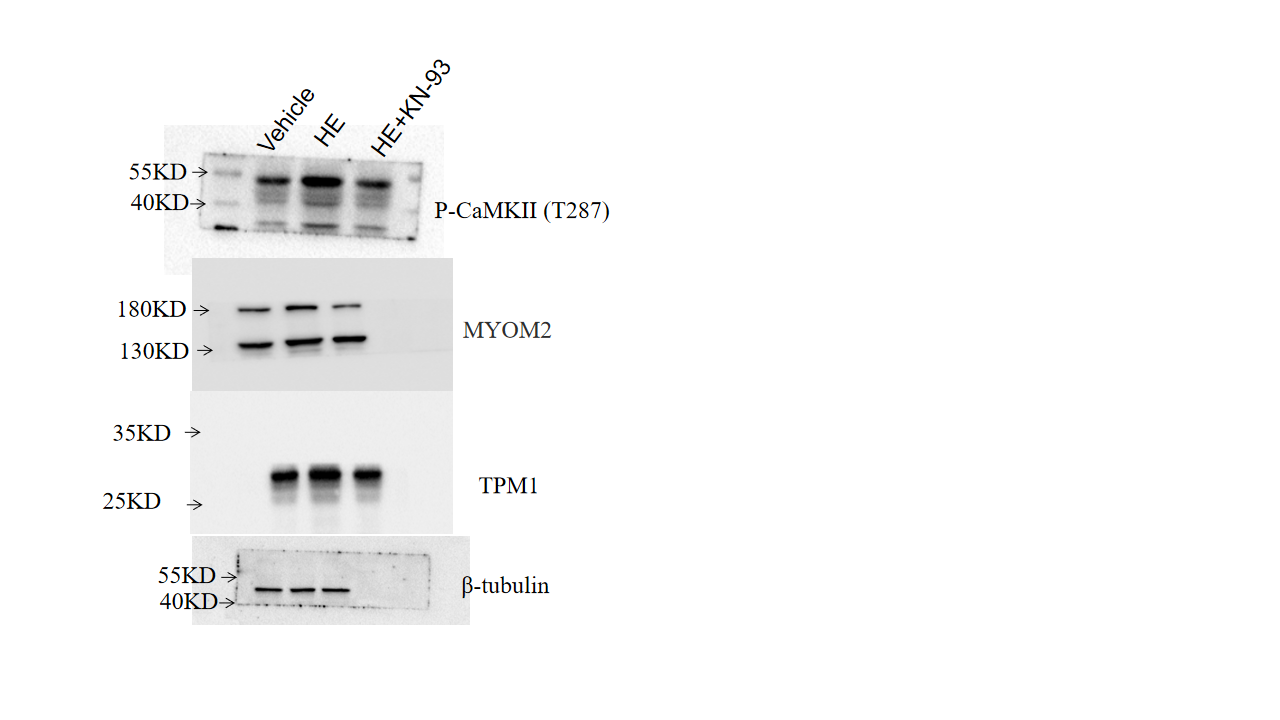

Supplement: Supplementary file 2 — Supplementary Figures. [file 41598_2023_32941_MOESM2_ESM.docx]
